# Supplementary figures and images for: Identification of significant gene and pathways involved in HBV-related hepatocellular carcinoma by bioinformatics analysis
Source: PeerJ. 2019 Jul 30;7:e7408. doi: 10.7717/peerj.7408 (PMC6677124; doi:10.7717/peerj.7408)

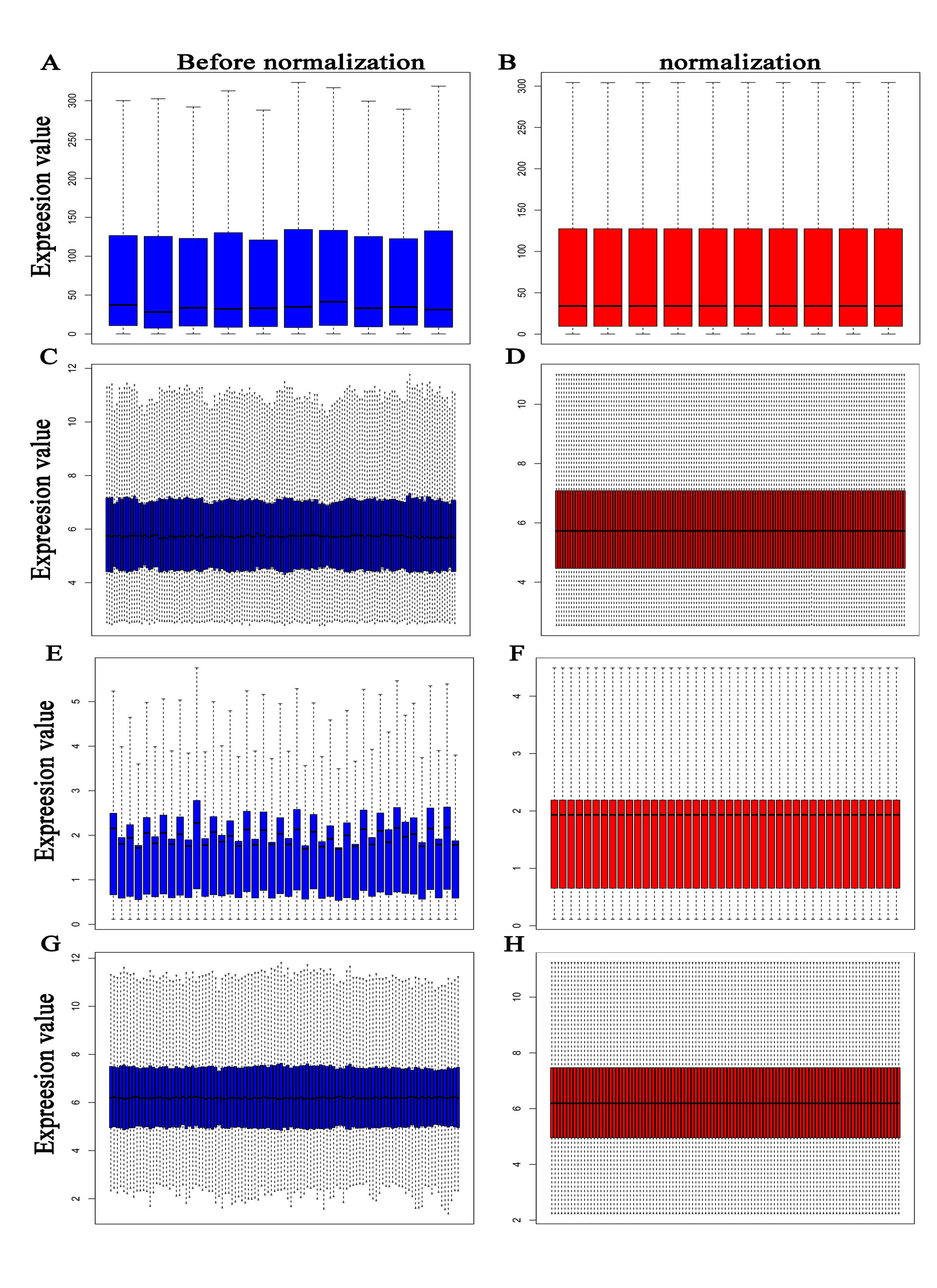

Supplement: Figure S1 — (A–B) Normalization of the GSE19665 data set. (C–D) Normalization of the GSE55092 data set. (E–F) Normalization of the GSE94660 data set. (G–H) Normalization of the GSE121248 data set. Blue represents data before normalization, and red represents data after normalization. [file peerj-07-7408-s001.jpg]

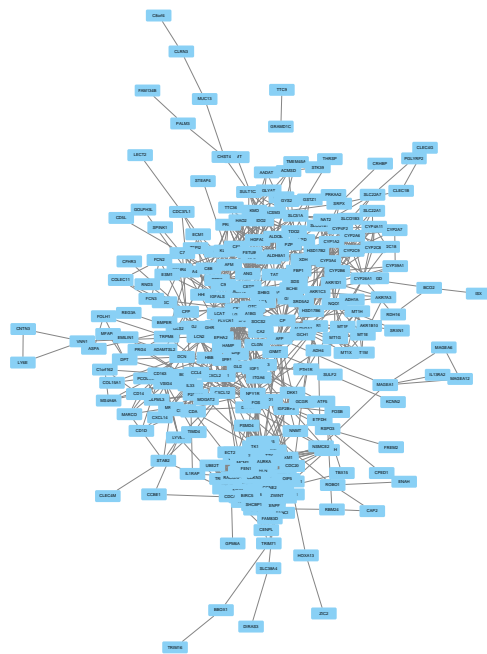

Supplement: Supplemental Information 2 — Table S1: The number of DEGs was identified in each database. TableS2: A total of integrated 341 DEGs were identified from the four datasets. Figure S2, Tables S3, S4: PPI network of the 341 DEGs was established by STRING. Table S5, S6 :The two most significant modules of the PPI network. [file peerj-07-7408-s002.zip › Figure S2.pdf]
